# Electroencephalogram-Based Analysis of Monomodal and Multimodal Interaction in Mixed Reality Games

**DOI:** 10.3390/s26123690

**Published:** 2026-06-10

**Authors:** Pratheep Kumar Paranthaman, Nikesh Bajaj, Logan LaMont

**Affiliations:** 1 Department of Computer Science, Elon University, Elon, NC 27244, USA; loganlamont25@gmail.com; 2 School of Physical and Chemical Sciences, Queen Mary University, London E1 4NS, UK; nikesh.bajaj@qmul.ac.uk

**Keywords:** mixed reality, extended reality games, electroencephalogram, interaction modalities, EEG analysis, NASA task load index, Self-Assessment Manikin, player experience

## Abstract

Mixed reality (MR) technologies enable users to experience computer-generated content within the physical environment through spatial computing and head-mounted displays. By supporting real-time interaction through speech, gesture, gaze, and movement, MR offers new opportunities for game design beyond productivity and educational applications. However, relatively few studies have examined interaction modalities in MR games. In this paper, we present the design and deployment of four MR games on the Microsoft HoloLens 2: three that use monomodal input (speech, gaze, or gesture) and one that uses multimodal input (speech, gaze, and gesture). We conducted a study with ten participants and evaluated player experience using subjective self-reports of task load, emotional engagement, and comfort alongside objective measures, namely brain activity data collected with a five-channel electroencephalogram (EEG) device. Our preliminary findings suggest two clusters of interaction modalities based on subjective measures, a pattern that is also reflected in the objective EEG measures. Our analysis combining subjective and EEG data indicates that interaction modality influences task load and emotional engagement. Additionally, our functional connectivity analysis showed links in activity across the prefrontal, temporal, and occipital brain regions for different input modalities in the MR games.

## 1. Introduction

Mixed reality (MR) systems blend digital content with the physical environment and allow virtual objects to respond to real-world surfaces and spatial layouts. Modern MR systems such as the Microsoft HoloLens 2, Apple Vision Pro, and Meta Quest 3 increasingly support natural, multimodal inputs, like gaze, eye tracking, speech, and hand gestures [1,2,3]. These input channels provide more intuitive and expressive interaction than traditional controllers, and they create opportunities for new forms of play that span the physical and virtual space. At the same time, these modalities differ in their motor demands, attentional requirements, and sensitivity to tracking or recognition errors. Understanding how different MR input modalities influence players’ workload, comfort, and engagement is therefore essential for designing MR gameplay that remains comfortable and enjoyable over longer sessions.

Despite rapid advances in MR hardware, current applications remain concentrated in productivity, learning, simulation, and visualization, with comparatively fewer examples of fully developed MR games [4]. Designing for MR requires not only traditional gameplay considerations but also spatial design and mapping game mechanics into physical environments, blending virtual actions with real-world movement, and managing how much cognitive and physical effort these experiences impose. MR headsets also introduce practical challenges like limited field of view, device weight, unfamiliar interaction techniques, and the potential for discomfort such as nausea, dizziness, or eye strain [5,6].

In terms of analyzing user experience, many HCI and game studies have incorporated the electroencephalogram (EEG) to gather insights into interaction from the perspective of brain activity [7,8,9]. EEG records moment-to-moment brain activity and has been used to examine how different interfaces and game mechanics influence users’ mental workload, attentional engagement, motor preparation, and affective state. For example, prior work has used EEG to distinguish more and less demanding game levels to classify players’ expertise and engagement [10]. In virtual reality (VR), EEG has also been used to estimate cognitive workload while participants perform tasks in interactive environments, showing that different workload levels can be distinguished from EEG patterns [11]. Recent studies have applied VR and EEG in game-related contexts by using EEG to monitor mental workload and engagement during VR games, relate game difficulty and flow to EEG measures, and compare brain activity with VR and conventional 2D displays [12,13]. Also, prior work has typically assessed MR and VR interaction using subjective self-report measures, including standardized questionnaires for workload, user experience, and cybersickness [14,15,16]. Self-report measures capture participants’ subjective impressions of workload, user experience, and comfort, but combining them with objective measures such as EEG can add additional depth to evaluation and analysis. When subjective ratings are examined alongside EEG, researchers can explore which neural patterns are associated with particular reported experiences and identify distinctions that may not be visible in questionnaire scores alone. For example, feeling positive, exerting effort, and performing well may all contribute to a higher workload rating (in environments like VR/MR), yet EEG patterns can reveal that these experiences align with different regional activity and functional mapping of brain regions on specific tasks in gameplay.

Despite growing interest in using EEG to study interactive systems, there is still limited work combining EEG with MR games to examine how different input modalities shape interaction and experience over time. Existing work rarely compares multiple MR interaction modalities within the context of moment-to-moment gameplay while integrating both subjective self-reports and EEG measures.

To address these aspects, we combined subjective self-reports with EEG measures to examine how different MR input modalities influence player experience during gameplay. In this study, we define monomodal interaction as gameplay that relies on a single form of input, such as gaze, speech, or gesture. We define multimodal interaction as gameplay that requires the coordinated use of multiple input channels within the same game.

We designed four MR games for the Microsoft HoloLens 2, and we centered each game on a distinct interaction modality. Three games used monomodal interaction through gaze, speech, or gesture, and one game used multimodal interaction by combining gaze, speech, and gesture within the same gameplay experience. We recruited ten participants to play all four games. We collected measures of emotional engagement using the Self-Assessment Manikin (SAM), workload using the NASA Task Load Index (NASA TLX), comfort and user experience using the Virtual Reality Neuroscience Questionnaire (VRNQ), and brain activity using a five-channel EEG device.

Primarily we investigated the following three research questions:

*RQ1: How do monomodal and multimodal MR interactions differ in emotional engagement and affective responses?*

*RQ2: How do different interaction modalities (speech, gaze, gesture, multimodal) affect task load, comfort, and user experience in MR games?*

*RQ3: How do the objective EEG measures of workload, engagement, and emotion align with subjective self-reports (SAM, NASA TLX, and VRNQ)?*

Our preliminary findings revealed three primary outcomes. First, all modalities produced positive valence, but gesture and multimodal interaction increased arousal, while gaze increased dominance. Second, gesture and multimodal input increased perceived workload, yet all modalities remained comfortable with low levels of virtual reality-induced symptoms and effects. Third, EEG patterns differed across affect, effort, and performance, and multimodal gameplay showed broader prefrontal–temporal and prefrontal–occipital connectivity in brain activity. These neural patterns indicate that MR interaction engages multiple underlying processes and that interaction complexity is reflected not only in local power changes but also in large-scale cortical coordination.

We believe this comparison of monomodal and multimodal MR games contributes to games research in extended reality (XR), interaction modality studies, EEG analysis for interactive systems, and human–computer interaction.

## 2. Related Work

### 2.1. Mixed Reality Games and Player Experience

Unlike traditional computer games, MR games do not rely on a single predominant input device such as a keyboard, mouse, or gamepad. Instead, MR games place interaction within the player’s surrounding environment and distribute input across the body and space. Players often walk within the physical environment; turn, reach, and perform mid-air gestures; and in some cases coordinate these movements with gaze and speech. Compared to VR games, which immerse players in a fully virtual scene and largely detach interaction from their actual surroundings, MR games must balance immersion with continuous awareness of physical obstacles, bystanders, and room layout. So, spatial design and interaction modality play a central role in shaping how players experience presence, comfort, and agency in MR play.

Prior research has explored how these embodied and spatial characteristics influence MR gameplay. Studies of immersive mixed reality street play show that head-mounted MR experiences affect how people interact and collaborate in public environments [14]. These findings informed guidelines for responsible and inclusive MR design and emphasized that public MR play remains underexplored. Similarly, investigations of high- and low-intensity MR games on HoloLens 2 demonstrate that spatial layout and movement demands directly influence cognitive load, emotional response, and overall experience [4].

Broader research examining MR systems emphasizes the importance of aligning virtual immersion with real-world awareness. Particularly, the research study by [17] suggests that this balance can enhance engagement while reducing side effects commonly associated with fully immersive VR, such as motion sickness and isolation. At the same time, analyses of MR hardware and interaction affordances show that device capabilities directly constrain and enable gameplay design, shaping what kinds of spatial and interactive experiences are feasible [18]. Empirical comparisons across platforms further indicate that MR gameplay can increase enjoyment and satisfaction relative to mobile experiences, even when task performance differs [19]. However, usability evaluations of MR games also reveal persistent challenges, particularly in presence and interaction fidelity, where limitations in hand tracking or spatial alignment can disrupt immersion [20]. In our research study, we also encountered several challenges in the gesture-based gameplay, particularly in recognizing specific gesture patterns in the MR experience.

Research on educational MR games further illustrates the potential of situated and collaborative experiences. For example, a mixed reality learning environment enabled first-year engineering students to navigate a digitally augmented production setting and practice automation concepts through game-based tasks [21]. Work on ecological validity likewise demonstrates that immersion increases when virtual elements meaningfully align with physical objects, whereas misalignment reduces coherence and engagement [22].

### 2.2. Interaction Modalities in Mixed Reality

Prior research in mixed and augmented reality has explored diverse input techniques, including gaze-based selection, mid-air gestures, and speech commands. Interaction fidelity has been shown to influence perceived presence and behavioral outcomes, indicating that modality choice is central to immersive experience rather than merely a technical decision [1].

Gaze-based interaction has received particular attention in MR systems. A gaze-aware learning system developed for the Microsoft HoloLens 2 demonstrated that integrating attention feedback through eye tracking improved learning outcomes [23]. Similarly, research conducted by [24] proposed a taxonomy of AR/MR interaction modalities and discussed design considerations for gaze input, including challenges related to targeting precision and calibration. Hybrid techniques combining gaze selection with hand confirmation illustrate how gaze can be integrated into multimodal interaction schemes [25]. Collectively, this research suggests that gaze supports efficient, hands-free interaction but depends heavily on careful interface design.

Gesture-based interaction has likewise been examined extensively. Studies of on-body micro-gestures in MR gameplay report that while traditional controllers provide higher speed and precision, gesture input offers an engaging and socially adaptable alternative [26]. A review of hand gestures in AR/MR applications [27] examined the role of gesture-based interaction across diverse immersive contexts and concluded that gestures can support natural and intuitive human–computer interaction by enhancing spatial awareness and, in certain cases, reducing cognitive load during complex tasks. These findings position gesture as a meaningful and embodied input modality within immersive systems.

Beyond individual modalities, multimodal interaction has gained attention for combining the strengths of gaze, gesture, and speech. Comparative analyses of tangible, collaborative, and hybrid interfaces suggest that integrating multiple input channels can enhance engagement and contextual immersion while potentially increasing coordination demand [28]. Such multimodal systems leverage the strengths of individual modalities like gaze for targeting, gesture for manipulation, and speech for command-based input, while potentially introducing additional coordination demands and workload. Blending virtual immersion with ongoing awareness of the real environment can increase engagement while helping to mitigate common VR side effects such as motion sickness, eye strain, and feelings of isolation [17].

### 2.3. EEG Measures in Extended Reality Games

Player experience in extended reality (XR) environments has traditionally been evaluated through subjective self-report measures assessing workload, affect, immersion, and comfort. While these instruments provide valuable insight into perceived experience, they rely on retrospective reflection and may not fully capture dynamic cognitive and emotional fluctuations during active interaction. Several studies have integrated VR and EEG in gaming [29], architectural design [30], hospital and clinical settings [31], motion sickness analysis [32], and therapeutic interventions [33]. The researchers in these works used EEG to observe cognitive workload, emotional state, or discomfort in immersive environments and to evaluate or adapt corresponding VR experiences.

Application-focused research embeds EEG into specific XR contexts. For example, exergame studies report elevated frontal theta activity, corresponding to increased cognitive engagement [34]. VR multitask experiments associate the preparatory frontal theta with successful attentional responses during gameplay [35]. MR medical education systems demonstrate the feasibility of combining EEG with HoloLens-based applications despite hardware constraints [36]. Sensorized head-mounted displays integrating EEG and additional biosignals further show that neural and physiological markers correlate with presence, immersion, and emotional states during commercial VR gameplay [37].

More recent methodological work formalizes synchronization pipelines for EEG, eye tracking, and XR events, identifying technical challenges such as electrical noise, constrained electrode placement, and latency alignment [38]. However, most existing EEG-XR research focuses on controlled tasks or fully immersive VR rather than spatially embedded MR gameplay. Few studies systematically compare interaction modalities within MR games or analyze moment-to-moment coupling between subjective experience and neural activity during specific in-game actions.

### 2.4. Research Gaps and Study Motivation

Across the literature, MR gameplay research has emphasized spatial design, usability, and subjective experience, while interaction modality research has largely focused on isolated tasks or single applications. Physiological measurement studies in XR demonstrate the feasibility of EEG integration but predominantly center on VR or controlled experimental paradigms rather than spatially embedded MR games. There remains limited work that (1) systematically compares monomodal and multimodal interaction modalities within multiple fully implemented MR games, (2) integrates EEG with HoloLens-based gameplay under realistic movement conditions, and (3) triangulates subjective workload and affect with objective neural measures at the level of moment-to-moment interaction. Our study addresses these gaps by combining Microsoft HoloLens 2 gameplay with five-channel EEG recordings across four interaction modality-driven MR games. Through analysis of subjective and neural data, this work contributes to the understanding of how interaction modality shapes workload, comfort, and emotional engagement in mixed reality play.

## 3. Design and Implementation of MR Games

We designed four MR games to investigate different interaction modalities. Each game was deployed on the Microsoft HoloLens 2, and we limited each play session to under ten minutes to reduce potential discomfort from wearing both the headset and the EEG device. The monomodal games each emphasized a single primary interaction modality as the core mechanic. We created three such games that used gaze, speech, or gesture as their only input. We also built one multimodal game that combined gaze, speech, and gesture. Together, these games allowed us to examine how different interaction modalities influence player workload, comfort, and emotional experience.

### 3.1. Monomodal Games

#### 3.1.1. Alien Tower Defense (Gaze-Based Game)

**Figure 1:**
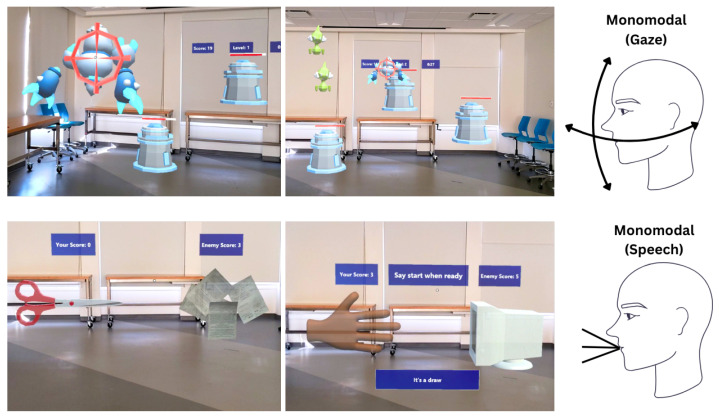
(**Top**) Gaze-based Alien Tower Defense (ATD) gameplay on HoloLens 2, where players target aliens using a head-directed gaze, indicated by the red reticle; Level 2 introduces green friendly allies that the player should not destroy. (**Bottom**) Speech-based gameplay, where players provide verbal commands and receive immediate round feedback.

Alien Tower Defense (ATD) is a gaze-based tower defense game. The goal of the player is to defend three towers from waves of incoming aliens (see Figure 1). The game uses the gaze input as its only interaction. When players wear the HoloLens 2, a red reticle appears at the center of the display and aligns with their gaze direction. Using this reticle, players must aim at aliens by slightly moving their head up, down, left, or right. When the reticle remains fixed on an alien for two seconds, the alien is destroyed. The game provides an audio cue after each successful hit. Each alien moves toward one of the three towers and attacks it. A tower is destroyed when its health reaches zero, and the player loses the game if all three towers are destroyed in a level.

In ATD, we have two levels, and each level lasts 80 s. Level 1 introduces waves of aliens that increase in number and speed. Level 2 adds friendly allies who appear randomly on the screen. These allies do not move. If the player destroys a friendly ally, they lose five points. To complete level 2 successfully, players must earn at least 25 points and keep at least one tower alive.

We designed ATD to include only gaze as the sole input modality. During early testing, we noticed that players may experience neck strain due to frequent head movement. To reduce strain, we adjusted the alien size and movement speed. For this, we included larger and slower targets to support smoother gaze tracking and less head rotation. We also positioned the towers four meters from the player to align with the HoloLens 2 field of view. This placement would allow players to maintain a stable head posture during gameplay.

#### 3.1.2. Rock, Paper, Scissors (Speech-Based Game)

In this game, we adapted the classic Rock–Paper–Scissors into a speech-based experience within the MR environment. Players provide speech commands to select their move by saying “rock,” “paper,” or “scissors” on their turn (see Figure 1). The computer then reveals its selection, and the winner of the round is determined based on the standard Rock–Paper–Scissors rules. The winner earns one point per round, and the game ends when the first player reaches seven points.

As in the ATD game, the user interface is positioned 4 m from the player’s starting point to ensure the entire scene remains within the field of view. When the player is ready, they say “start” to begin a round, which triggers a 5-s countdown displayed in front of them. During this interval, the player must speak their chosen option; if they fail to respond within 5 s, the round resets and a new 5-s timer begins. After the player issues a command, the corresponding 3D model is activated to represent their choice. The computer then selects its move, and the system displays the outcome of the round along with the current scores for both the player and the computer.

#### 3.1.3. Broken in Space (Gesture-Based Game)

**Figure 2:**
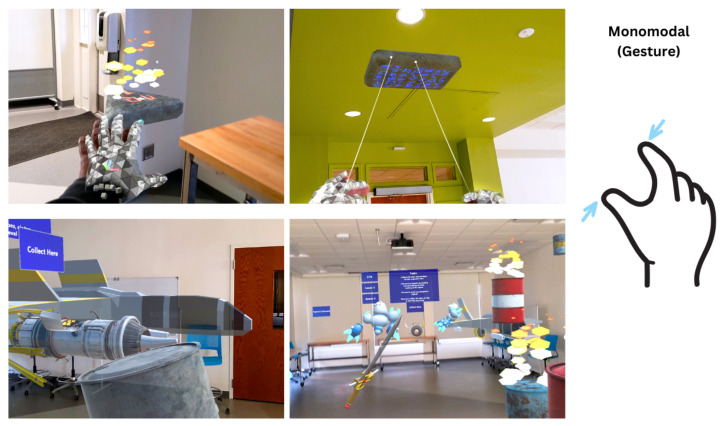
Gesture-based Broken in Space gameplay on HoloLens 2. (**Top left**) Open-palm gesture to extinguish fire on a rune and (**Top right**) distance-grab gesture pulling a rune. (**Bottom**) Level 3 scene with moving enemies, including barrels and a spaceship.

Broken in Space (BiS) is a gesture-based puzzle game that requires players to move around the space and solve small puzzles using gestures such as open palm, closed fists, and pinching. The player’s goal in the game is to fix a broken spaceship by completing three gesture-driven levels. Each level lasts 210 s, and the difficulty increases across levels (see Figure 2).

In Level 1, the player collects runes scattered in space to activate a sword. They approach a rune, perform a pinch gesture to collect it, then walk to the sword and release the rune onto it. The player must place 7–8 runes. Some runes are on fire; the player first shows a stop gesture (open palm) to extinguish the fire and then uses the pinch gesture to collect the rune. Some runes appear high above the player and require a distance grab gesture. The player raises both hands in front of the HoloLens visor to activate two beams, aims at a distant object, pulls it closer, and then uses the pinch gesture to grab the rune. After placing the required number of runes, the sword activates, and the player can carry it using the pinch gesture.

In Level 2, the player repairs the spaceship engine by collecting scrap metal from space. The collection mechanic mirrors Level 1 but introduces static enemies that the player must avoid. If the player collides with a static enemy, the game ends. After placing the required scrap metal on the engine, the player progresses to the final level. In Level 3, the player collects oil barrels and drops them onto the spaceship engine. This level introduces moving enemies that the player must avoid. The player can use the sword obtained in Level 1 in both Levels 2 and 3 to defeat enemies. Once the engine is repaired, the player places it back onto the spaceship model in the game space to complete the game. This game encourages the use of multiple gestures and continuous movement within the play area.

### 3.2. Dragon Slayer—Multimodal Game

The primary design goal of this game is to examine how players coordinate speech, gaze, gesture, and physical movement within a single MR gameplay scenario. The game adopts a first-person shooter-style perspective and combines the three interaction modalities explored in the monomodal games: speech, gaze, and gesture. The player’s objective is to defeat a dragon using a virtual sword.

**Figure 3:**
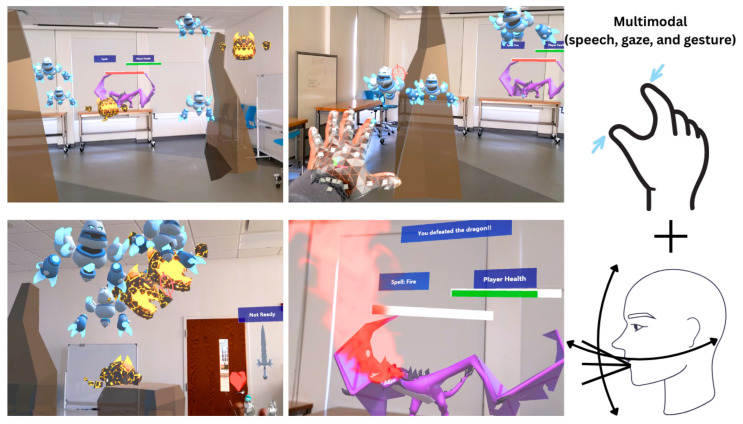
Screenshots from the multimodal Dragon Slayer gameplay on Microsoft HoloLens 2. (**Top left**) The game environment showing spatial navigation elements, including rocks used for cover and roaming fire and ice enemies. (**Top right**) The player using an open palm gesture to halt an enemy, followed by an air-tap gesture to destroy it. (**Bottom**) Sword activation and the final encounter where the player defeats the dragon.

To activate the sword, the player must first defeat fire and ice enemies in the environment. The player issues speech commands such as “fire” or “ice” to activate the corresponding spell. After activating a spell, the player aims at an enemy using a gaze-aligned reticle and performs an air-tap gesture by pinching their index finger and thumb of their right hand. This interaction sequence mirrors the aiming and shooting actions commonly found in traditional first-person shooter games (see Figure 3).

After the player defeats all fire and ice enemies, the sword becomes available. The player walks toward the sword, collects it using a pinch gesture, and carries it to the dragon. The player then drops the sword onto the dragon to deal damage. Each successful hit removes one third of the dragon’s health, which is displayed on a health bar. The player must strike the dragon three times to complete the game. After each hit, the sword resets and a new set of fire and ice enemies appears. During combat, the dragon periodically emits fire attacks. The player must physically move behind rocks in the environment to take cover. As the game progresses, the dragon increases the frequency of its attacks, raising the difficulty level. Health pickups appear when the player defeats fire or ice enemies and allow the player to restore lost health. The gameplay requires players to coordinate speech, gaze, gesture, and full-body movement throughout the experience.

## 4. Methods

### 4.1. Participants

**Figure 4:**
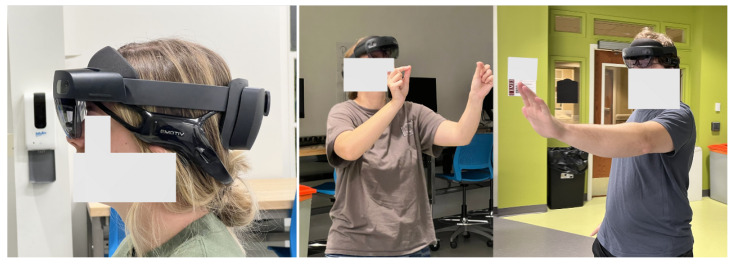
Participants interacting with the games. (**Left**) Participant wearing Microsoft HoloLens 2 and the Emotiv Insight five-channel EEG headset. (**Center**) Participant performing a two-handed distance-grab gesture during gameplay. (**Right**) Participant performing an open-palm gesture during gameplay. All participants wore the HoloLens 2 and EEG headset throughout the experiment.

We recruited 10 participants (7 male, 3 female) from a university population. The mean age was 21.7 years (SD = 2.00). Four participants had previously used the HoloLens 2. Six participants had played mixed reality games before, and nine had played virtual reality games. Regarding experience with MR devices, five participants reported that they had rarely used such devices (once or twice), four had never used them, and one reported moderate experience (5–10 uses) (see Figure 4).

### 4.2. Apparatus

We developed all the MR games using the Unity game engine and the Mixed Reality Toolkit (MRTK) SDK. Within the MRTK SDK [39], we used components for speech recognition and hand gesture input, and for tracking users’ gaze, we implemented a custom reticle system. Before development, we conducted a bodystorming and physical role-playing process to explore interaction constraints and game scenarios [40]. This process helped us define spatial boundaries, spawn locations, and item placement in the play area. We measured the physical space and mapped those measurements into Unity to position virtual objects consistently with the real environment.

After implementing the games in Unity, we deployed them on the Microsoft HoloLens 2. For data collection, we used an Emotiv Insight 5-channel EEG headset [41]. During the user study, participants first wore the Emotiv Insight headset and then the HoloLens 2. We selected the 5-channel configuration to ensure a comfortable fit and avoid mechanical interference with the HoloLens 2, which we encountered with 14- and 32-channel variants. The raw EEG data were streamed wirelessly to the Emotiv Pro software on a desktop computer, and we later exported these recordings for processing and analysis.

### 4.3. Procedure

Each participant completed a pretest questionnaire to report basic demographics and prior experience with MR and VR devices. Participants then played four games corresponding to distinct interaction modalities: three monomodal conditions (gaze, speech, gesture) and one multimodal condition (combining gaze, speech, and gesture), each implemented as a separate game with similar objectives and difficulty. Because each condition maps directly to a specific game, we use the terms “game” and “interaction modality” interchangeably in the remainder of this paper. These four games were assigned in a random order. After playing each game, participants completed three post-task surveys. These included the Virtual Reality Neuroscience Questions (VRNQ) to assess comfort and physical symptoms in the MR environment, the Self-Assessment Manikin (SAM) to measure emotional experience (valence, arousal, dominance), and the NASA Task Load Index (NASA TLX) to evaluate perceived workload.

**Figure 5:**
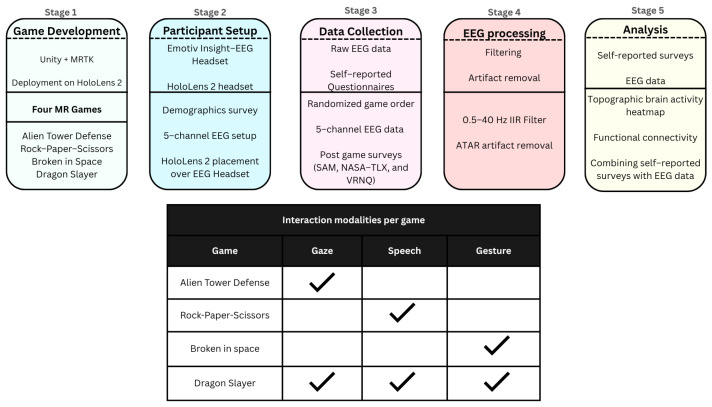
Overview of the research study pipeline. This table summarizes the interaction modality associated with each game.

Each game session had a 7-min time limit to minimize discomfort from wearing both the HoloLens 2 and EEG headset. Actual playtime ranged from 4–7 min per game. Also, we observed that the gesture-based game often approached the maximum time due to its emphasis on physical movement and gesture interactions like distance grabbing and precise object placement. In total, the study sessions lasted 45–70 min. At the end of the study sessions, the participants received a $10 Amazon gift voucher for their time (see Figure 5).

### 4.4. Measures

We collected both self-reported and EEG measures. After each game, participants completed the SAM [42], the NASA-TLX [43], and selected items from the VRNQ [44]. SAM captured valence, arousal, and dominance, each rated on a 9-point scale from 1 (very low) to 9 (very high). NASA-TLX captured six workload dimensions: mental demand, physical demand, temporal demand, performance, effort, and frustration; each rated on a 10-point scale. From the 20-item VRNQ, we used only the user experience and virtual reality-induced symptoms and effects (VRISE) parts, totaling 10 items. The user experience subscale comprised five items (immersion, enjoyment, graphics quality, sound quality, and overall technology quality), each rated from 1 (extremely low) to 7 (extremely high). The VRISE subscale comprised five items (nausea, disorientation, dizziness, fatigue, and instability), each rated from 1 (extremely intense feeling) to 7 (absent). In total, participants answered 18 self-report items after each game.

Also, during gameplay, we recorded raw EEG using an Emotiv Insight 5-channel headset with electrodes at AF3, AF4, T7, T8, and Pz. The headset transmitted raw EEG signals wirelessly to Emotiv Pro, running on a separate computer. For each participant and each game condition, we exported the corresponding EEG file for subsequent preprocessing and analysis.

### 4.5. Data Processing and Analysis Methods

In this section, we explain the preprocessing steps for subjective data (SAM, NASA TLX and VRNQ). In addition, we also describe the methods used for EEG data processing, conducting analysis and extracting results.

#### 4.5.1. Preprocessing of Subjective Ratings and Visualization

**Figure 6:**
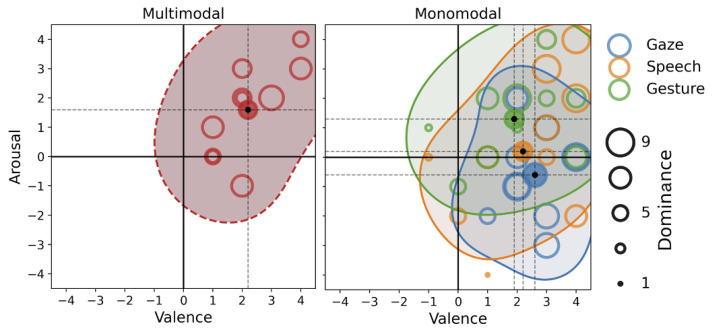
SAM Comparison: Comparison between monomodal and multimodal with SAM ratings. Valence and arousal are on x and y axis, and dominance is represented as size of circle.

**Figure 7:**
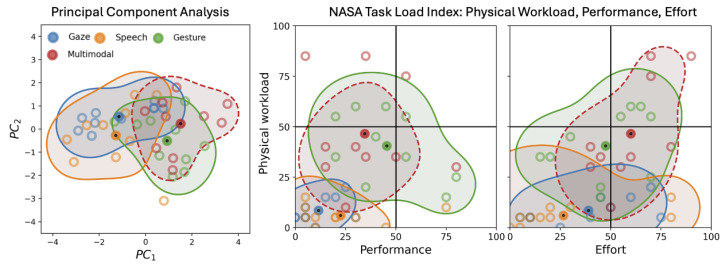
NASA TLX Comparison: (**Left**) Principal Component Analysis of NASA Task Load Indexes (**Right**) of physical workload with performance and effort across all games.

For SAM, the valence and arousal ratings were changed from 1–9 to −4–4, keeping the dominance the same as 1 to 9. The contour plots for SAM and NASA TLX are generated using the Kernel Density Estimation (KDE) technique using Gaussian Kernel [45]. Specifically, for SAM, KDE is generated using valence and arousal values (see Figure 6). For NASA TLX, to avoid creating contour plots for many combinations of two indicators (e.g., (6×6−6)/2=15), we first applied Principal Component Analysis (PCA) to reduce its 6 dimensions to 2 dimensions, and applied a contour plot on the first two principal components (i.e., $PC_{1}$ and $PC_{2}$). We also show contour plots for the three most differentiating indicators, namely physical workload, performance, and effort, as shown in Figure 7.

**Figure 8:**
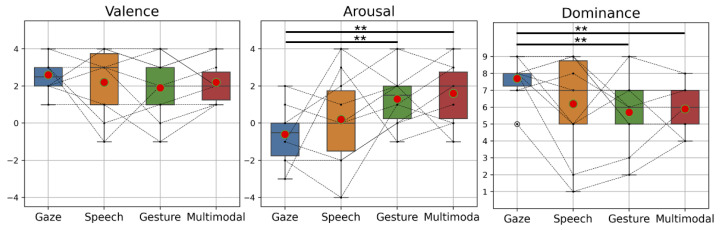
SAM Comparison: Comparing valence, arousal, and dominance across different games. A significant difference between two groups is indicated with a horizontal line and ** (** =p<0.01).

**Figure 9:**
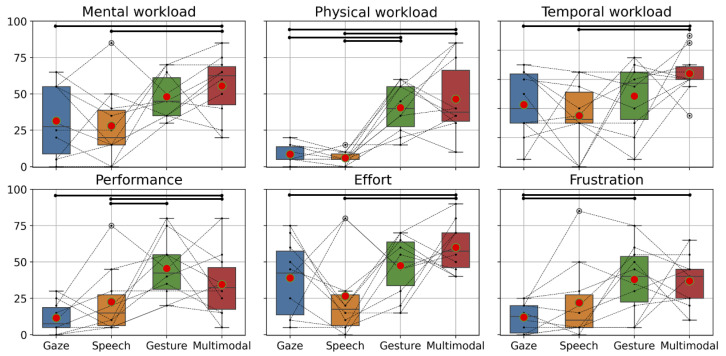
NASA Comparison: Comparing all six NASA Task Load Indexes across different games; mental, physical, temporal workload, performance, effort, and frustration. A significant difference between two groups is indicated with a horizontal line (p<0.01).

**Figure 10:**
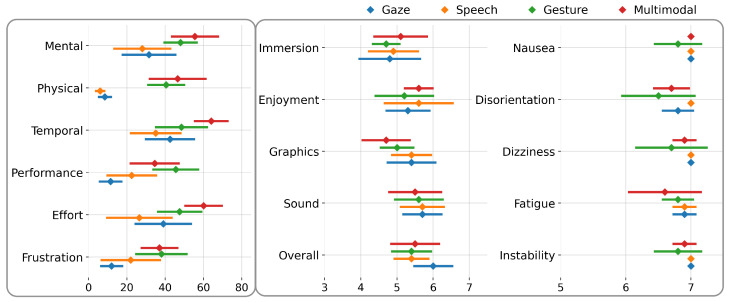
NASA Task Load Index and VRISE rating: Mean with 95% confidence interval across games.

Finally, to show the differences among games with respect to SAM and NASA TLX, we used box-and-whisker plots (see Figure 8 and Figure 9). As the VRNQ data were less variable and differentiating among the games, we used a mean μ with 95% confidence ($\mu \pm 1.96\sigma_{e}$) interval plot to show the VRNQ data, in addition to NASA TLX, as shown in Figure 10.

#### 4.5.2. Statistical Analysis

To compare two groups statistically, we applied a paired parametric *t*-test if a normality test was passed; otherwise, a non-parametric Wilcoxon signed-rank test was applied. In all cases with a significant difference, the normality test was passed. For SAM and NASA TLX, all the pairs that were found significantly different (p<0.05) are indicated in Figure 8 and Figure 9 with horizontal lines.

#### 4.5.3. Preprocessing of EEG Recordings and Power Computations

The raw EEG signals were first processed with a highpass filter with an IIR filter of order 5 with a cut-off frequency of 0.5 Hz, followed by a lowpass filter with a cut-off frequency of 40 Hz. To remove artifacts from the EEG recordings, we used the ATAR algorithm with soft-thresholding mode and tuning parameter β=0.01, as well as the db3 wavelet [46].

After preprocessing, we computed the power (in dB) for each electrode in five frequency bands, namely δ: 0.5–4 Hz; θ: 4–8 Hz; α: 8–16 Hz; β: 16–32 Hz; and full-band *f*: 1–40 Hz. We computed the average (mean) and variation (standard deviation) of the power in a frequency band over the entire recording for each electrode and denoted them as $P_{k}^{\sigma }$ and $S_{k}^{\sigma }$, respectively. Here, σ indicates the frequency band (i.e., σ∈δ,θ,α,β,f), and *k* is the electrode number (i.e., k∈1,2,3,4,5) for electrodes AF3,AF4,T7,T8, and Pz. For example, $P_{1}^{\alpha }$ and $S_{1}^{\alpha }$ are the average power and standard deviation of the power within the α band over the entire EEG recording at electrode AF3. To capture the dynamics of brain activity, we also computed the average power of an electrode for a frequency band over a small duration using a moving window of 3 s with an overlap of 1 s. We denote the dynamic power of an electrode *k* in the σ frequency band as $P_{k}^{\sigma }\left(t\right)$.

#### 4.5.4. Topographical Map of EEG

**Figure 11:**
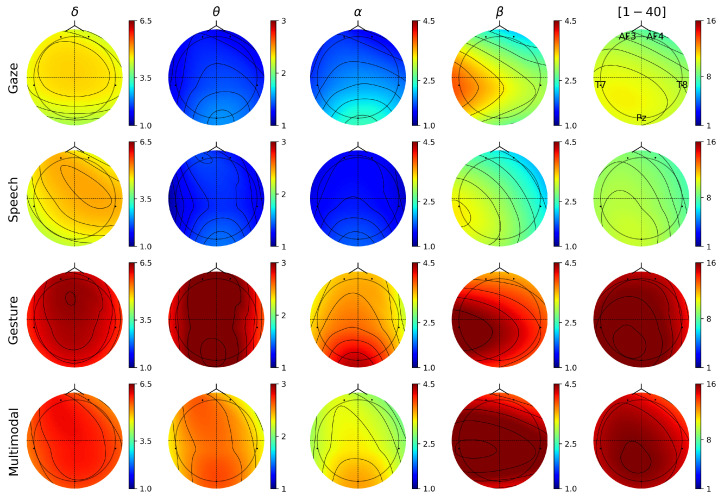
Topographic brain activity heatmaps showing mean EEG power values across participants in different frequency bands for each interaction modality. Red and orange colors indicate higher power values, while blue and green colors indicate lower power values. Electrode names and locations are shown in the top-right map as a reference.

**Figure 12:**
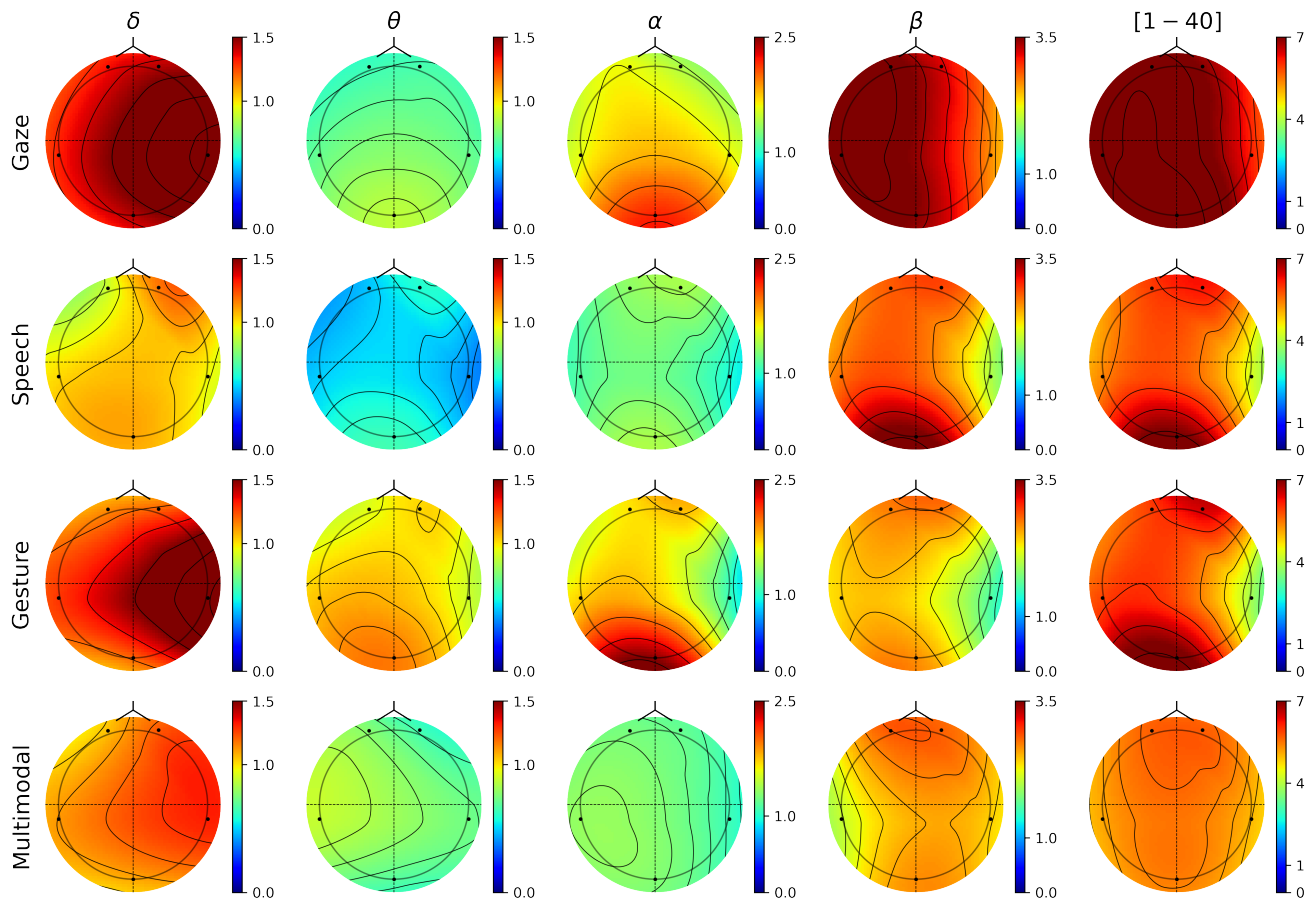
Topographic brain activity heatmaps of the standard deviation of EEG power values across participants for each interaction modality (gaze, speech, gesture, multimodal) and frequency band (δ, θ, α, β, and full band 1–40 Hz). Higher standard deviation values are shown in red/orange and lower values in blue/green, with color scales held constant within each band.

The topographic map of EEG is a heatmap displaying values (average power value) as colors for respective brain regions. Using the SpKit toolkit [47], we displayed the average and variation in mean power across all the participants for each game and each frequency band, as shown in Figure 11 and Figure 12, respectively. For instance, the average and the standard deviation of the mean power of electrode AF3 $P_{1}^{\alpha }$ is computed across all the participants. An example of a topographic map with the names of electrodes is shown in Figure 11 in the top-right corner.

#### 4.5.5. Functional Connectivity Analysis

The functional connectivity analysis establishes the associations (dependency) between different regions of the brain. It allows us to analyze how different parts of the brain work together under given conditions. There are various approaches to establish the functional connectivity [48]; we used the temporal change in the average power of electrodes. To start, if the $i^{th}$ electrode is significantly associated with the $j^{th}$ electrode within a frequency band σ in the presence of other electrodes (also called conditional association), we employed multiple regression on the dynamic average power $P_{i}^{\sigma }\left(t\right)$, as follows:

(1) $$P_{i}^{\sigma }\left(t\right)=w_{0}+\sum_{j\neq i}w_{j}P_{j}^{\sigma }\left(t\right)\;\;\;\mathrm{where}\;\sigma \in \left\{\delta ,\theta ,\alpha ,\beta ,f\right\}$$

If the coefficient $w_{j}$ reveals a value with *p*-value p<0.05, we consider that as electrode $P_{i}^{\sigma }\left(t\right)$, which is significantly associated with $P_{j}^{\sigma }\left(t\right)$. We repeat this for each electrode with each frequency band until all the significant associations are extracted for each player. While computing Equation (1), we excluded the first and last 5 windows (i.e., first and last 5 s) of the recordings. For example, if $P_{AF3}^{\alpha }\left(t\right)$ has a significant association with $P_{T7}^{\alpha }\left(t\right)$ and $P_{Pz}^{\alpha }\left(t\right)$, the brain region’s left prefrontal lobe is associated with the left temporal lobe and occipital lobe.

**Figure 13:**
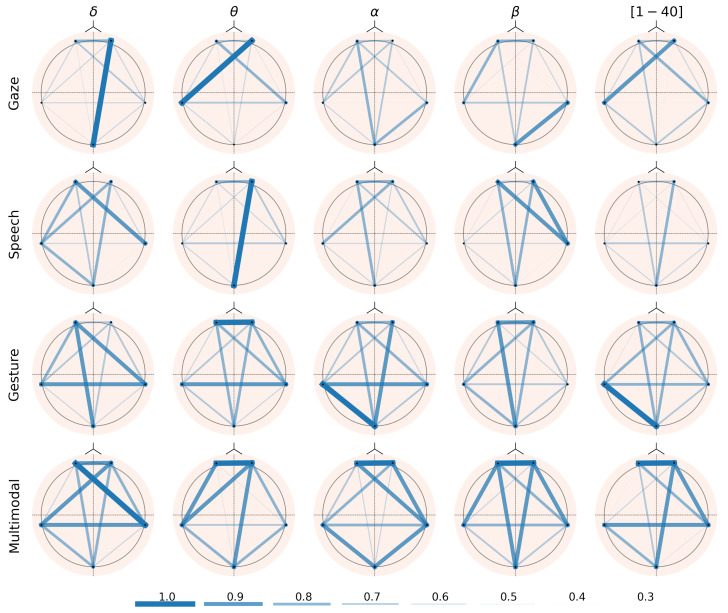
This figure shows the average functional connectivity across participants for each interaction modality and frequency band. The thickness of each line indicates the proportion of participants in whom that connection is present: the thickest lines (value 1.0) indicate connections present in all participants (100%), whereas connections present in fewer than 50% of participants are drawn very thin.

To compute the common functional connectivity for a game withing a frequency band, we averaged all the significant connections across participants. The averaged functional connectivity for each game within each frequency band is shown in Figure 13. The percentage of participants that were discovered to have a particular connection is shown as thickness of the line, which is indicated at the bottom of the figure. The thickest line with value 1 indicates that such a significant connection was found in all the participants, and less than 50% (<0.5) is an almost thin and invisible line.

#### 4.5.6. Combining Subjective (SAM, NASA TLX, VRNQ) and Objective Data (EEG)

To establish the relationship between the subjective and objective measures, we employed multiple regression on normalized values. We used the subjective measures, $Y_{M}$ (one of the SAM, NASA and VRNQ measures), the average power $P_{k}^{\sigma }$ and the standard deviation $S_{k}^{\sigma }$ as follows:

(2) $$Y_{M}=c_{0}+\sum_{k=1}^{5}a_{k}P_{k}^{\sigma }+\sum_{k=1}^{5}b_{k}S_{k}^{\sigma }\;\;\;\mathrm{where}\;\sigma \in \left\{\delta ,\theta ,\alpha ,\beta ,f\right\}$$

The coefficients $a_{k}$ and $b_{k}$ (with respect to the objective measures) indicate the relationship of the measure $Y_{M}$ with the average power and the standard deviation of the power, respectively. The coefficient $a_{k}$ corresponds to average power and coefficient $b_{k}$ corresponds to the standard deviation of power. The direction of association (positive or negative) depends on the sign of the coefficients. For example, a coefficient with a significant value of $a_{1}=0.1$ indicates a positive association with an average power of the 1st electrode, AF3, and $b_{3}=-0.2$ indicates a negative association with a standard deviation of power of the 3rd electrode T7.

**Figure 14:**
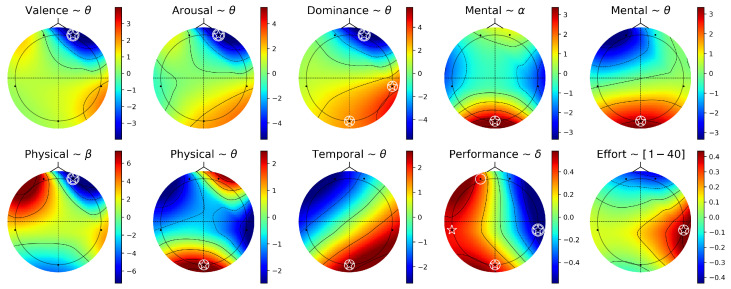
Association between EEG activity and SAM/NASA-TLX ratings obtained using multiple regression. Each topographic map shows regression coefficients linking a questionnaire scale to the band-limited mean EEG power at the five electrodes, with blue indicating negative associations and red/yellow indicating positive associations. White stars mark electrodes where the association with mean power is significant (p<0.05). White circles mark significant associations with the standard deviation of power: a single circle indicates a positive association, and a double circle indicates a negative association at that location/EEG electrode.

**Figure 15:**
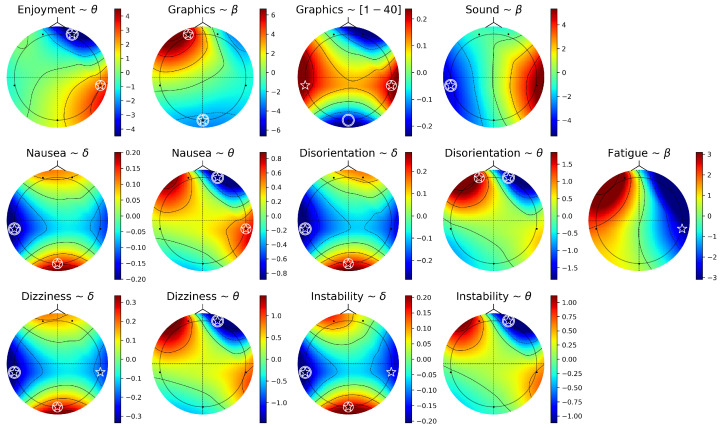
Association between EEG activity and VRNQ ratings obtained using multiple regression. Each topographic map shows regression coefficients linking one VRNQ item (enjoyment, graphics, sound, nausea, disorientation, fatigue, dizziness, instability) to mean EEG power at the five electrodes, with blue indicating negative associations and red/yellow indicating positive associations. White stars mark electrodes where the association with mean power is significant. White circles mark significant associations (p<0.05) with the standard deviation of power: a single circle indicates a positive association, and a double circle indicates a negative association with power variability at that location.

The results of the association established using multiple regression are displayed as topographical maps, as shown in Figure 14 and Figure 15. For example, Figure 14 in the top left shows the association established for valence within the θ-band, with the average power and standard deviation of power. The topographic map is generated only with the values of average power $a_{k}$, and the coefficients with significant values (p<0.05) for $a_{k}$ are indicated with a white star. As we avoided displaying topographical maps for the standard deviation $b_{k}$, we indicated significant values with white single circles for a negative association and double circles for a positive association.

## 5. Results

### 5.1. Emotional Engagement in Monomodal vs. Multimodal Games

Figure 6 (left) shows that most of the valence and arousal values of the multimodal responses are in upper-right quadrant of the affective space, indicating that they are primarily clustered in the positive-valence and moderate-to-high arousal region. The mean values of valence and arousal for the multimodal case are centered in the upper-right quadrant. Dominance values varied across participants, as reflected by their differences in circle size.

For the monomodal conditions, the patterns in Figure 6 (right) indicate that emotional responses differed across the interaction conditions. While valence remained positive for gaze, speech, and gesture, arousal levels varied more across modalities. Gesture-based interaction elicited comparatively higher arousal responses, whereas gaze-based interaction tended to correspond with lower arousal for several participants. Speech-based interaction showed intermediate and more widely distributed arousal values.

Figure 8 provides a statistical comparison of valence, arousal, and dominance across modalities. For valence, the box plots show overlapping distributions with no significant differences between gaze, speech, gesture, and multimodal interaction. For arousal, statistically significant differences (p<0.01) were observed between gaze and multimodal interaction, as well as between gaze and gesture interaction. These initial results show higher arousal levels for multimodal and gesture conditions relative to gaze. No significant differences were observed between speech and the other modalities. For dominance, statistical significant differences emerged between gaze and multimodal interaction and between gaze and gesture interaction. Gaze-based interaction showed higher dominance values relative to the gesture and multimodal conditions. Other pairwise comparisons did not reach statistical significance.

### 5.2. Task Load, Comfort, and User Experience Across Modalities

Similar to box plot of SAM, Figure 9 shows the NASA TLX responses for comparing across different modalities. For the mental, physical, temporal, and effort scales, multimodal interaction demonstrated a significantly higher workload compared to gaze and speech. Gesture-based interaction also showed an elevated workload relative to gaze and speech, particularly for physical demand. Across these dimensions, no significant differences were observed between gaze and speech, and gesture and multimodal often exhibited overlapping distributions.

For performance, statistically significant differences (p<0.05) were observed between multimodal and speech, multimodal and gaze, and speech and gesture, as indicated in Figure 9. No significant differences were found between gaze and speech or between multimodal and gesture. This pattern further reflects the clustering of gaze and speech at lower workload levels and gesture and multimodal at higher workload levels. Frustration was observed to be higher in the gesture modality compared to all the others, and was significantly higher than gaze. Frustration in the multimodal case was also significantly higher than gaze. Again, gaze and speech did not significantly differ, and gesture and multimodal showed overlapping distributions.

Furthermore, the PCA analysis of NASA TLX across interaction modalities revealed two clusters, separating gaze and speech from gesture and multimodal interaction, based on the contour plot of $PC_{1}$ and $PC_{2}$, as shown in Figure 7. Gaze and speech clustered toward lower values of $PC_{1}$, whereas gesture and multimodal clustered toward higher values of $PC_{1}$.

The second principal component ($PC_{2}$) explained additional variance but did not produce a comparable separation between modalities. Variability along $PC_{2}$ appeared within modalities rather than between modality groups. In Figure 7, the middle and right panels show the contour plots between physical workload and performance and effort, respectively. As can be observed in this figure, the gesture and multimodal interactions were associated with higher physical workload and effort levels, whereas gaze and speech remained concentrated at lower levels. These projections align with the $PC_{1}$ separation observed in the PCA plot.

Figure 10 presents the NASA TLX subscale means with 95% confidence intervals across interaction modalities. Similar to box plot and PCA analysis, Figure 10 shows overlapping of gaze and speech and a comparatively lower workload across the scales. However, gesture and multimodal interaction cluster at higher levels. In several dimensions, particularly physical workload, effort, and temporal demand, the separation between these modality groupings is reflected by limited overlap in confidence intervals. The 95% confidence interval visualization supports the presence of two consistent workload profiles: a lower-demand profile (gaze and speech) and a higher-demand profile (gesture and multimodal).

For the VRNQ, we used ten items: five items captured overall experience (immersion, enjoyment, graphics, sound, and overall quality), and five items captured virtual reality-induced symptoms and effects (VRISE), and these included nausea, disorientation, dizziness, fatigue, and instability. Figure 10 presents the VRNQ means with 95% confidence intervals across interaction modalities. For the first five VRNQ items, all modalities showed mean ratings in the upper range of the scale, with overlapping confidence intervals between all interaction modalities. This pattern is consistent with broadly positive evaluations of game quality and immersion across modalities. For the VRISE-related items, all modalities showed mean ratings very close to 7, an indication of absence with substantial overlap in the confidence intervals. These values correspond to low levels of reported discomfort and suggest that VRISE remained minimal for all four games.

### 5.3. Comparing EEG Measures with Self-Reported Experience

Figure 11 presents the topographic brain activity heatmap with the distribution of mean power across participants within each frequency band (δ, θ, α, β, and full-band *f* 1–40 Hz) for the three monomodal games and one multimodal game.

Across all bands, gaze and speech interaction show comparatively lower power, with predominantly blue to light-green values over most brain regions. The brain activity in the θ and α bands shows much lower values for gaze and speech compared to gesture and multimodal. The gesture interaction exhibits elevated power in every band, with more extensive yellow-to-red/orange regions indicating increased activity in δ, θ, α, β, and full bands. The multimodal condition shows a similarly elevated profile, with widespread red/orange areas and particularly strong δ and full-band power. The mean EEG power distributions reveal a modality-dependent pattern; in this case, gaze and speech are associated with lower overall power (more blue/green) in the frontal and central regions across all analyzed bands, whereas gesture and multimodal interaction show higher and more spatially extensive activation (more yellow/red/orange) in the δ, θ, α, β, and full-band ranges.

Figure 12 shows the standard deviation (variation) of the average EEG power across participants for each modality and frequency band. The lower standard deviation values are shown in blue and higher values in red/orange. In the δ band, gaze and gesture gameplay exhibit higher variability than speech, indicating larger between-participant differences in low-frequency activity for these modalities. For gaze, variability is also elevated in the β and full-band *f* ranges, suggesting that power levels in these bands differ more across players than in the corresponding speech and multimodal conditions. Within the θ band, gesture interaction shows slightly higher variability than the other three games, pointing to greater individual differences in θ responses during gesture-based gameplay.

Figure 13 shows the average functional connectivity across participants for each gameplay condition and frequency band. Each line represents a connection between two electrodes, reflecting a functional link between corresponding brain regions (as observed from EEG). Line thickness indicates the proportion of participants exhibiting that connection. As noted in the legend, connections present in fewer than 50% of participants appear very thin, whereas the thickest lines (average = 1.0) indicate connections consistently observed across all participants.

Gaze and speech conditions exhibit fewer and more spatially constrained functional connections compared to gesture and multimodal interaction. Gesture and multimodal conditions show denser and more distributed connectivity patterns across the frontal, temporal, and occipital regions. In the gaze condition, selective but consistent connections are observed. For example, in the δ band, a relatively strong connection appears between Pz and AF4, indicating a link between the occipital and right prefrontal regions. In the θ band, connectivity between the left temporal region and the right prefrontal region is present across all participants (average = 1.0). A similar cross-regional pattern is also visible in the full-band *f* representation. These patterns suggest focused inter-regional coordination rather than widespread connectivity. Speech interaction shows more distributed connections than gaze, particularly between the occipital and prefrontal regions and between the occipital and temporal regions. These connections appear across multiple frequency bands, although they are generally less extensive than those observed in gesture and multimodal gameplay.

The gesture and multimodal conditions display the most extensive and consistent connectivity patterns. In gesture gameplay, strong connections appear between the left and right prefrontal regions in the θ band. Additional consistent links are observed between the occipital and left temporal regions in the α band and full-band *f* representation.

Multimodal interaction exhibits strong connectivity overall. Notably, consistent connections between the left and right prefrontal regions are present across all frequency bands. In the δ band, a strong connection between the right temporal and right prefrontal regions is observed across participants. Across bands, multimodal gameplay shows multiple cross-regional connections, indicating widespread and coordinated brain activity. The functional connectivity results show modality-dependent brain connectivity patterns. Gaze and speech interactions are associated with more localized and selective connectivity patterns, whereas gesture and multimodal gameplay are associated with broader and more consistently shared coordination across participants.

In Figure 14, we first examined how SAM and NASA-TLX ratings related to EEG power at each electrode and frequency band. The rightmost map in the bottom row shows that effort is positively associated with mean power and negatively associated with standard deviation at the right temporal electrode T8 in the full *f* 1–40 Hz band, indicating stronger but more stable activity in this region for effort. Valence, arousal, and dominance all exhibit a common pattern (negative association with mean and negative association with standard deviation) in the θ band at the right prefrontal electrode AF4. Dominance additionally shows positive associations with both mean power and standard deviation at a posterior electrode PZ, indicating a broader θ band involvement than valence and arousal.

For the NASA-TLX workload dimensions, mental demand is positively associated with both mean power and standard deviation at the occipital electrode Pz in the α and θ bands. Physical demand shows a distinct frontal pattern in the β band, where a higher physical workload is linked to lower mean power and a lower standard deviation at AF4. In the θ band, physical and temporal demands exhibit positive associations with mean power and standard deviation at Pz, suggesting that increased workload and self-reported performance coincide with enhanced occipital θ activity. Performance further shows positive associations with mean power at Pz and T7 and with standard deviation at AF3 and Pz, but negative associations with both mean power and standard deviation at T8, pointing to a dissociation between the left/posterior and right temporal contributions to perceived performance.

Similar to Figure 14, Figure 15 presents topographical maps that depict how VRNQ ratings are associated with EEG power across brain regions and frequency bands. From Figure 15, the power in the θ band shows a similar association pattern for disorientation, nausea, instability, and dizziness: these VRNQ items are negatively associated with right prefrontal activity and positively associated with left prefrontal activity. The power in the δ band also exhibits a common pattern for disorientation, nausea, instability, and dizziness, with positive associations at occipital regions and negative associations at the left temporal lobe. Enjoyment is positively associated with right temporal activity and negatively associated with right prefrontal activity in the θ band. Graphic quality is associated with the occipital as well as the left and right temporal regions in the full band *f* (1–40), and sound quality is associated with β band activity in the left temporal lobe.

## 6. Discussion

### 6.1. Interpreting Emotional Engagement Across Interaction Modalities (RQ1)

Our RQ1 addresses how monomodal and multimodal MR interactions differ in emotional engagement and affective responses. Our results from Section 5.1 indicate that all four interaction modalities produced positive valence, showing that each game maintained overall enjoyment. However, the modalities differed in arousal and dominance, as shown in Figure 8. This pattern suggests that within our task and session limits, adding or changing modalities did not make the experience feel unpleasant, even when it increased arousal or reduced dominance.

Arousal and dominance differentiated the conditions in ways that mapped onto the underlying game mechanics. Gesture and multimodal interaction elicited higher arousal than gaze. Both games required players to move through space, coordinate hand actions, and respond to dynamic threats under time pressure. These mechanics likely increased activation due to movement and simultaneous task demands. Because valence remained positive, this higher arousal reflects engagement rather than negative stress. Gaze-based interaction produced lower arousal and higher reported dominance. The gaze game required players to remain relatively stationary and use head movements to target moving enemies. This monomodal input provided a direct mapping between gaze and action and may have supported a stronger sense of control. The reduced physical demand and absence of modality coordination likely contributed to a calmer experience.

Speech-based interaction produced intermediate arousal and moderate dominance. The turn-based structure required players to provide discrete verbal commands within a time window and then wait for the system’s response. Some players may have experienced strong control over the timing of their input, while others may have perceived reduced agency due to the structured, non-continuous interaction format. Dominance varied more widely in the gesture and multimodal conditions. These games required players to coordinate locomotion and gesture execution, and, in the multimodal case, there were also scenarios in which the players needed to use speech commands for switching between the spells within a single session. Such coordination demands may have influenced dominance differently across individuals. Some players may have experienced this combination as flexible and empowering, while others may have experienced it as complex to manage within the limited exposure time.

Overall, our preliminary findings for RQ1 show that monomodal and multimodal interactions did not differ in pleasantness but differed in the arousal and dominance they produced. Interaction modality and associated mechanics may function as design levers that can shape emotional activation and perceived control in MR gameplay.

### 6.2. Interpreting Workload, Comfort, and User Experience Across Modalities (RQ2)

Our RQ2 focuses on how speech, gaze, gesture, and multimodal interaction affect task load, comfort, and user experience in MR games. The NASA TLX results (as outlined in Section 5.2) show two consistent workload profiles: a lower-demand profile for gaze and speech, and a higher-demand profile for gesture and multimodal interaction. This pattern suggests that modality choice shapes how demanding players perceive MR gameplay, even when game content and session length remain comparable. The mechanics in the gesture-based and multimodal games increased physical demand and effort. The need to manage locomotion, object interaction, and timed responses likely contributed to their elevated perceived workload. In contrast, the gaze and speech-based games relied on monomodal input and limited spatial movement. These mechanics reduced physical demand and simplified coordination requirements, which corresponded to lower perceived workload. The performance ratings reflect similar structural differences. The multimodal and gesture gameplay required simultaneous monitoring of threats, timing actions, and coordinating multiple inputs. These layered demands may have influenced performance and made tasks feel more demanding. In comparison, gaze and speech interactions involved more constrained and predictable input structures, which may have supported a more manageable experience. Frustration patterns suggest that workload does not map directly onto comfort. Gaze interaction involved sustained precision, and small misalignments could interrupt actions. Gesture interaction required accurate hand tracking, distance grabs, and spatial positioning. These modality-specific sensitivities may have contributed to frustration independently of overall workload level. Comfort therefore appears to depend not only on aggregate demand but also on the stability and responsiveness of the interaction channel.

The VRNQ results refine this picture by showing that despite these workload and frustration differences, participants still reported a high overall experience score and low levels of VRISE symptoms across all modalities. Immersion, enjoyment, audiovisual quality, and overall ratings remained in the upper range of the scale for each game, suggesting that players generally perceived the experiences as polished and engaging. This consistency may partly reflect the technological aspects of mixed reality: players can always see the physical world, which reduces visual occlusion and may support comfort and a stable sense of presence. It may also reflect our content choices, since all four games used a similar low-poly visual style and shared constraints on asset size to keep each build compact for HoloLens 2 deployment. Because we held art style and visual complexity relatively constant across the games, the VRNQ scores likely capture differences driven more by interaction modality than by graphical fidelity, and different patterns might emerge if future work systematically varied art styles or visual density.

At the same time, nausea, disorientation, dizziness, fatigue, and instability scores stayed close to the “absent” end of the scale, indicating that even the more demanding gesture and multimodal conditions did not translate into notable simulator sickness or instability within our session length and task structure. This combination, higher workload without strong VRISE, suggests that the increased demand in gesture and multimodal interaction manifested primarily as effortful, active play rather than physiological discomfort. Again, the near absence of discomfort across all games may relate to the characteristics of mixed reality: players retained visual access to their real environment and did not experience full-field visual replacement, which can contribute to cybersickness in virtual reality. We anticipate that running the same interaction designs in a fully virtual setup could produce a wider range of VRISE responses, particularly for movement-intensive and multimodal conditions. Comparing VR and MR versions of these games using VRNQ would therefore be a valuable direction for future work. Prior work [49] that investigated VR and MR in a physical therapy context reported higher immersion and enjoyment in VR but slightly greater comfort in MR, which aligns with our observation that MR can support demanding and engaging gameplay while keeping VRISE symptoms low.

Lastly, the PCA results still indicate a dominant workload gradient across modalities, but our emphasis here is less on clustering and more on what drives this gradient. The pattern appears to reflect increasing physical involvement and multimodal coordination: as interaction shifts from stationary monomodal input toward movement-intensive multimodal mechanics, then perceived demand increases. The VRNQ outcomes indicate that this gradient in demand can coexist with consistently positive experience ratings and minimal adverse effects when the MR content, duration, and hardware setup are controlled.

Our findings for RQ2 show that the choice of interaction modality may shape perceived workload through its specific action and coordination demands, but these differences did not lead to reduced comfort or higher VRISE scores within the scope of our study. Gesture and multimodal control felt more effortful yet remained enjoyable in our MR setting. This suggests that under controlled task duration and visual complexity, it is possible to vary and even combine input modalities without necessarily compromising user comfort. However, the generalizability of this pattern likely depends on factors such as session length, visual load, and hardware stability, which future work should examine further.

### 6.3. Alignment of EEG and Subjective Measures (RQ3)

Our RQ3 examines how objective EEG measures of workload, engagement, and emotion align with subjective self-reports. The mean-power topographies (outlined in Section 5.3) reveal a clear modality-dependent pattern: gesture and multimodal interaction produced broadly elevated δ, θ, α, β, and full-band *f* activity across the frontal, parietal, temporal, and occipital regions, whereas gaze and speech elicited lower and more spatially constrained activation. This contrast is consistent with EEG research showing that sensorimotor and prefrontal regions exhibit stronger and mid-frequency activity when tasks involve motor planning, visuospatial coordination, or continuous movement [50]. The elevated frontal θ observed for gesture and multimodal conditions aligns with evidence tying the frontal-midline θ to cognitive control, task difficulty, and mental workload across a range of visuomotor and decision-making tasks. The lower θ in gaze and speech conditions is therefore compatible with their simpler, monomodal control demands and reduced motor complexity [50,51].

The spatial distribution of α and β activity reinforces this pattern. Gesture and multimodal interaction showed increased α and β power over the occipital cortex, consistent with evidence that these rhythms reflect planning, visuomotor integration, and modulation of motor drive during reaching and grasping. In contrast, gaze- and speech-based control, which minimize proprioceptive and motor involvement, produced correspondingly lower activity in these regions [52,53,54]. The mean power and variability patterns indicate that modalities with greater motor involvement and coordination demands elicit higher and more widely distributed cortical activation, whereas simpler monomodal inputs elicit weaker activation that is confined to fewer regions.

As shown in Figure 13, the functional connectivity patterns also align with workload and interaction complexity aspects. Gaze and speech produced relatively sparse focal networks, with a few stable links between occipital, temporal, and right-prefrontal sites, consistent with focused visuomotor and attentional demands in simpler control schemes [55]. Gesture and multimodal interaction, by contrast, yielded denser connectivity between the bilateral prefrontal, temporal, and occipital regions across multiple bands, resembling the more integrated networks reported in multi-tasking and complex visuomotor paradigms under a higher mental workload [56,57,58]. The presence of prefrontal–temporal and prefrontal–occipital couplings during multimodal gameplay suggests that coordinating several input channels involves widespread control and sensory systems rather than a single localized region. This pattern indicates that interaction complexity in MR games may be reflected not only in local power changes but also in broader cortical coordination.

The regression analyses (presented in Figure 14 and Figure 15) show that SAM and NASA-TLX ratings do not simply increase with overall EEG activity. Instead, each rating maps onto a small set of specific EEG patterns, in particular, brain regions. Higher valence, arousal, and dominance correspond to lower θ activity at the right-prefrontal site AF4, and dominance additionally involves θ activity at the posterior site Pz. This pattern is compatible with prior work showing that frontal asymmetry and midline θ/α/β power support emotion recognition and predict SAM-like ratings such as dominance [59]. It therefore suggests that feeling positive or in control in mixed reality may rely on a more efficient and less variable engagement of frontal and parietal control processes, rather than on uniformly stronger frontal activation. For the effort aspect, it shows an opposite profile: higher effort relates to stronger and more stable full-band activity at the right-temporal site T8, pointing to a key role of temporal regions in sustaining attention to audiovisual cues when participants feel they are trying hard.

In the NASA-TLX workload ratings, mental and temporal demand show increased α/θ activity at Pz, indicating that visual and attention-related processing becomes more engaged when the task feels mentally demanding or time-pressured [60]. Physical demand shows reduced β activity at AF4, consistent with frontal β decreasing as bodily or motor effort increases. Performance combines these effects: higher performance is associated with stronger δ activity at Pz and T7 but reduced activity at T8, suggesting that doing well depends more on effective visual–motor monitoring than on the sustained effort-related patterns expressed at the right-temporal sites. These factors highlight that EEG patterns indicate which brain regions support feeling positive, feeling effortful, and performing well during mixed reality gameplay, highlighting that subjective experience is underpinned by distinct neural patterns rather than a single global workload signal.

In summary, combining EEG measures with self-report data for RQ3 suggests that players’ ratings may not reflect a single global sense of workload. Instead, affect, effort, and performance appeared to be associated with distinct patterns of prefrontal, temporal, and occipital activity, and EEG analysis provided preliminary insight into the nuances of how players engaged with MR interaction.

### 6.4. Practical Constraints and Future Work

During development and deployment on the HoloLens 2, we encountered practical constraints that influenced our design decisions. Complex 3D models and high-resolution textures led to rendering instability and increased build size. To maintain stable performance and reliable deployment, we adopted a low-poly art style and kept each game compact in size. Lighting conditions and spatial mapping also influenced gameplay stability. Because MR relies on real-time environmental understanding, object placement and tracking were sensitive to ambient lighting and surface geometry. We therefore conducted testing in a fixed physical space to ensure consistent spatial calibration. Future work should investigate how these modalities perform in dynamic or uncontrolled environments where lighting and spatial layouts vary.

To minimize EEG artifacts and discomfort associated with movement and headset placement, we limited gameplay duration to less than ten minutes per condition. Longer sessions may change workload, comfort, and neural patterns, so extended play remains an important direction for future research. Our multimodal game emphasized high-activity, first-person style mechanics, which likely increased whole body movement and, in turn, may have introduced additional movement-related artifacts into the EEG signal despite artifact removal. Future studies can build on this work by examining how multimodal interaction functions in lower-intensity gameplay contexts, such as puzzle solving or strategic reasoning, which are cognitively demanding but physically low in activity.

Also, this study has limitations related to sample size and demographic diversity. With ten participants, the findings should be regarded as preliminary and exploratory. Additionally, the gender imbalance in the sample meant that potential differences across gender groups in MR interaction experience could not be examined. Future studies should recruit larger and more demographically diverse samples.

Additionally, each modality was implemented as a distinct game, a deliberate design choice to provide gameplay experiences that fully leverage each modality’s natural interaction affordances in Microsoft HoloLens 2. As a preliminary exploration, we adopted this approach to first understand how each modality performs within its game context before examining more controlled comparisons. However, game-specific content may have independently influenced results beyond the effect of modality alone, and future work should explore designs that isolate modality effects within a single game environment.

Although MR produced minimal virtual reality-induced symptoms and effects in our study, a direct comparison between VR and MR using identical game mechanics would provide deeper insight into whether MR inherently supports greater comfort under similar interaction demands. Finally, EEG recording in MR contexts introduces artifact challenges due to head movement, gesture execution, device weight, and device placement overlap with Hololens 2. Although we applied the ATAR algorithm [46] to clean baseline artifacts, continued refinement of artifact detection and removal techniques will be required to improve the reliability of neural measures.

## 7. Conclusions

In this study, we investigated how monomodal and multimodal interaction modalities shape player experience in MR games. We designed and deployed four MR games on the Microsoft HoloLens 2 and compared gaze, speech, gesture, and multimodal input using measures of task load, emotional engagement, comfort, and brain activity. Examining these modalities within a single experimental framework allowed us to compare how different forms of interaction relate to subjective experience and neural activity.

Prior work has more often combined EEG with VR than with MR gameplay, and has less frequently used EEG together with self-reports to evaluate MR interaction modalities. In our data, interaction modality was associated with differences in reported workload, emotional engagement, and neural patterns. While all conditions showed low levels of virtual reality-induced symptoms within the tested duration, EEG analyses indicated region-specific activity patterns linked to affect, effort, and performance; moreover, functional connectivity suggested broader coordination of brain activity during multimodal gameplay.

We believe that EEG can complement self-reports by providing additional information about underlying patterns that may not be visible in subjective ratings alone. At the same time, applying EEG in MR settings requires careful attention to headset configuration, data collection procedures, artifact handling, sensor placement, and session length, all of which affect signal quality and interpretation. Within these constraints, we see this work as an initial step toward analyzing MR input modalities using neural measures alongside participants’ subjective responses.

## Data Availability

The data presented in this study are available upon request to the corresponding author. Availability of specific materials may vary depending on the nature of the request.
